# Application of a Promising Bone Graft Substitute in Bone Tissue Regeneration: Characterization, Biocompatibility, and *In Vivo* Animal Study

**DOI:** 10.1155/2019/1614024

**Published:** 2019-10-31

**Authors:** Endang Wahyuningtyas, Ling-Chuan Hsu, Wen-Chien Lan, Shih-Cheng Wen, Keng-Liang Ou, Hsin-Hua Chou, Mao-Suan Huang, Erwan Sugiatno

**Affiliations:** ^1^Department of Prosthodontics, Faculty of Dentistry, Universitas Gadjah Mada, Yogyakarta 55281, Indonesia; ^2^School of Dentistry, College of Medicine, Taipei Medical University, Taipei 110, Taiwan; ^3^Department of Oral Hygiene Care, Ching Kuo Institute of Management and Health, Keelung 203, Taiwan; ^4^School of Dental Technology, Taipei Medical University, Taipei 110, Taiwan; ^5^Department of Dentistry, Taipei Medical University Hospital, Taipei 110, Taiwan; ^6^Department of Dentistry, Taipei Medical University-Shuang Ho Hospital, New Taipei City 235, Taiwan; ^7^Department of Prosthodontics, Faculty of Dentistry, Hasanuddin University, Makassar 90245, Indonesia; ^8^School of Dentistry, Health Sciences University of Hokkaido, Hokkaido 061-0293, Japan; ^9^3D Global Biotech Inc., New Taipei City 221, Taiwan; ^10^Dental Department of Wan-Fang Hospital, Taipei Medical University, Taipei 116, Taiwan; ^11^School of Oral Hygiene, College of Oral Medicine, Taipei Medical University, Taipei 110, Taiwan

## Abstract

The purpose of the present study was to investigate the effect of local hydroxyapatite (HA) combined with extracted sea cucumber (*Stichopus hermanni*) collagen as a promising bone graft substitute on bone remodeling. Fourier-transform infrared spectroscopy, X-ray diffractometry, transmission electron microscopy, 3-(4,5-dimethylthiazol-2-yl)-2,5-diphenyltetrazolium bromide (MTT) assay, and Sprague-Dawley rat model were used to characterize the microstructure, *in vitro* cytotoxicity, and *in vivo* bone-healing properties of the investigated biocomposite material. Analytical results found that the hydrothermal reaction-synthesized local HA had a hexagonal close-packed structure. The addition of extracted *S*. *hermanni* collagen did not influence the microstructure and functional groups of the local HA. Moreover, the MTT assay indicated that the investigated biocomposite material possessed a good *in vitro* biocompatibility. The *in vivo* animal study also revealed that the investigated biocomposite material exhibited the highest number of osteoblasts after 14 days of healing. Therefore, the results demonstrate that the local HA combined with extracted *S*. *hermanni* collagen could potentially enhance osteoblast formation in promoting bone healing and regeneration.

## 1. Introduction

The alveolar bone gives major support to the immediate denture. The alveolar bone will experience resorption by 40% to 60% after tooth extraction, which will affect the use of immediate denture [1]. The denture is not stable if it is not supported by the alveolar bone [2]. The wound healing after tooth extraction is very important, especially before denture insertion [3]. The main problem encountered after tooth extraction is bone damage. The bone damage physiologically is resorption of the bone caused by tooth extraction [4]. The graft materials are applied to close gaps in the bone defect after tooth extraction resulting in bone formation, which allows the implant and denture to be installed [5, 6]. To restore bone function properly, addition or replacement of tissue is needed for damaged bone tissue in order to repair the bone damage and increase the volume of the alveolar bone. Thus, the artificial bone graft materials are used to rehabilitate bone damage for facilitating bone regeneration [7–9].

Hydroxyapatite (HA) is a good biocompatibility biomaterial and does not cause negative reactions to the human body. New bone formation is promoted by HA through the osteoconduction mechanism [10, 11]. Collagen is a natural polymer widely used as bone substitute materials in tissue engineering and repair. Moreover, collagen is resorbed easily and degraded by the body and has a good ability to attach on cells [12, 13]. Parisi et al. [14] have reported that incorporation of collagen from marine sponges into HA (especially the one mimicking the composition of the bone (with 70% of HA and 30% of spongin)) can improve the performance of the graft for bone regeneration applications. The findings provided a new strategic development of the novel biocomposite material as the bone graft substitute.

Recently, medicinal and health beneficial effects of functional sea cucumbers have been validated via scientific researches [15–17]. Among them, *Stichopus hermanni* collagen is a product of *S*. *hermanni* by collagen extraction, which contains 80% collagen. It has also been demonstrated that the extracts derived from *S*. *hermanni* possess various medicinal values such as accelerating wound healing [18], protecting neuronal cells against injury [19], and stimulating growth in mesenchymal stem cells [20]. Our research team has reported that the local HA combined with *S*. *hermanni* collagen did not cause systemic toxicity on the liver and kidney of Sprague-Dawley rats [21]. It possessed good biocompatibility potential to be a bone graft substitute. Therefore, the aim of the present study was to synthesize a promising biocomposite material, local HA combined with extracted *S*. *hermanni* collagen, as a novel bone graft substitute for the rehabilitation of the bone. The research objective was to investigate the effect of the novel biocomposite material on the osteoblast number of bone remodeling *in vivo*. The research findings could provide new scientific information in the dentistry field.

## 2. Materials and Methods

### 2.1. Preparation of the Investigated Biocomposite Sample

The fabrication procedure of local HA was based on the previous study [22]. The local HA was synthesized using gypsum powder (Kulon Progo, Yogyakarta, Indonesia). A 20 g of gypsum powder was mixed with 800 mL of 1 M diammonium hydrogen phosphate solution. Subsequently, the mixture solution was heated at 100°C for 20 min on Pyrex glass by means of a microwave digestion system under an operating frequency of 2.45 GHz. After the hydrothermal reaction, the reacted sample was cleaned using distilled water and then dried thoroughly. The *S*. *hermanni* collagen was extracted from *S*. *hermanni* using chloroform and methanol in a ratio of 1 : 1 according to the previous research [23]. Finally, the investigated biocomposite sample was prepared in an optimal concentration of 65% local HA and 35% *S*. *hermanni* collagen.

### 2.2. Characterization Analysis

The model PerkinElmer Spectrum 100 Fourier-transform infrared spectroscope (FTIR; USA) was applied to characterize the functional groups of the investigated sample. The model Rigaku 2200 X-ray diffractometer (XRD; Japan) equipped with the Cu K*α*1 radiation source and model JEM-2010F transmission electron microscope (TEM; JEOL, Japan) were employed to identify the phase and crystallinity of the investigated sample. The operation of XRD was carried out at 50 kV and 250 mA. Moreover, the diffraction peaks from the XRD spectrum were identified according to the database of the Joint Committee on Powder Diffraction Standards. For TEM sample preparation, a 20 *μ*L of suspension from the investigated sample mixed with ethanol solution was pipetted onto a copper grid coated with the carbon film. Afterward, the grid was dried thoroughly in an electronic dry cabinet and then observed at an accelerating voltage of 200 kV.

### 2.3. *In Vitro* Cytotoxicity Evaluation

The Vero fibroblast cell line was used in the cytotoxicity evaluation by the 3-(4,5-dimethylthiazol-2-yl)-2,5-diphenyltetrazolium bromide (MTT) assay. Before evaluation, the investigated sample was sealed in the sterile pouch and sterilized by ethylene oxide (3M 8XL, USA). Then, the investigated sample was mixed with 1 mL of phosphate-buffered saline solution (pH 7.4; Gibco, Indonesia) in seven different doses of 0.015 mg/mL, 0.031 mg/mL, 0.062 mg/mL, 0.125 mg/mL, 0.250 mg/mL, 0.500 mg/mL, and 1.000 mg/mL as the testing solution. Subsequently, the Vero fibroblast cell suspensions with a density of 5000 cells/mL were pipetted per well in 96-well polystyrene plates. Then, the testing solutions (*n* = 10) were added to each well and cultured in an incubator at 37°C with 5% CO_2_ for 24 h, respectively. After the incubation period had elapsed, 50 *μ*L of MTT-labeled solution was added to each well, and the plate was incubated for a further 4 h to form the formazan solvent precipitates. The supernatant was aspirated, and cells were lysed with 150 *μ*L of dimethyl sulfoxide for 10 min in a 96-well plate to release the formazan. Finally, the model BioTek Epoch ELISA Reader (USA) with a wavelength of 550 nm was employed to measure the optical density (OD) value in each dose. The cells in the blank group (control group) were only exposed to the medium.

The average number of live cells in each dose was compared with that of the control group. The reduction of viability was calculated by equation (1) which represents the cell activity:

(1) $$\begin{array}{c} \text{viability }\%=\frac{{\text{OD}}_{550\text{c}}-{\text{OD}}_{550\text{d}}}{{\text{OD}}_{550\text{c}}}, \end{array}$$

where OD_550d_ is the mean value of the measured OD of the investigated group and OD_550c_ is the mean value of the measured OD of the blank (control group). The investigated group is considered to have an acute cytotoxic potential when the viability percentage is lower than 50% that of the blank group.

### 2.4. *In Vivo* Animal Study and Histological Analysis

Sixty male Sprague-Dawley rats (age: 3 months and weight: 250–300 g) were used and divided into three groups in the animal study. The surgery was done at Laboratorium Penelitian dan Pengujian Terpadu and Veterinary Histopathology Laboratory, Universitas Gadjah Mada. Before surgery, shaving on the field of surgery (femur) was done in all animals. A dose of 8 mg Ketalar per 100 g (animal weight) was adopted as anesthesia. Then, the skin was opened with a longitudinal cut on condyles of the rat's femur. The bone defect with a size of 3 mm × 3 mm × 2 mm was prepared using a fissure drill to remove bone debris. Subsequently, the investigated samples (group I: the investigated biocomposite material, group II: only extracted *S*. *hermanni* collagen, and group III: only normal collagen) were filled into the bone defect, respectively. After treatment, the wound of the operation was sutured using absorbable catgut thread (Safil® 4.0, B. Braun Surgical S.A., Spain). Finally, the animals were sacrificed on days 3, 7, 10, 14, and 28. The histological sample was prepared using fixing, decalcifying, paraffin wax embedding, ultramicrotome sectioning, and hematoxylin and eosin staining. Then, the stained slides of histological samples were observed and analyzed by means of a high-quality digital image capture pathology scanner (Aperio CS, Germany) under different magnifications.

### 2.5. Osteoblast Count

In this study, the amount of osteoblasts was utilized as a parameter to know the effects of three investigated samples as a bone substitution material on bone remodeling. Osteoblast count was determined using the trinocular microscope with a 400x magnification camera on five fields in each sample. Each field has a diameter of 0.65 *μ*m and an area of 1.99 *μ*m^2^.

### 2.6. Data Analysis

Statistical analysis of the data was performed using two-way ANOVA and continued with the LSD test in SPSS (*p* ≤ 0.05).

## 3. Results

### 3.1. Chemical Bonding States and Microstructural Characteristics

**Figure 1:**
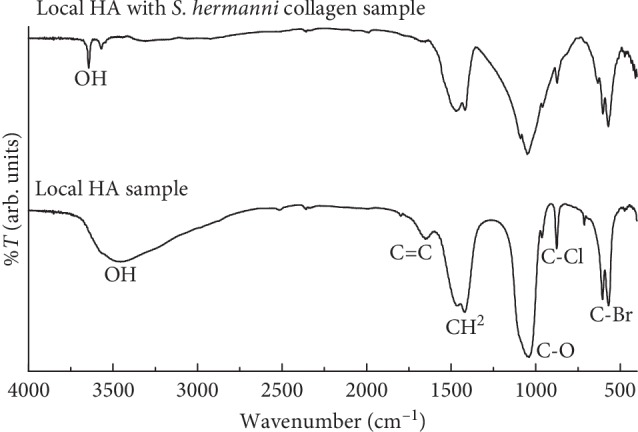
FTIR spectra of the local HA with and without *S*. *hermanni* collagen samples.

Figure 1 shows the FTIR spectra taken from the local HA with and without *S*. *hermanni* collagen samples. Clearly, the functional groups of OH, C=C, CH^2^, C-O, C-Cl, and C-Br were found in both samples. The OH functional group at wavenumber ∼3600 cm^−1^ belongs to the carboxylic acid OH stretch in the local HA sample. As the *S*. *hermanni* collagen added in the local HA, the functional group of water OH stretch can be detected at wavenumber ∼3700 cm^−1^. This feature is probably due to the addition of high-water solubility *S*. *hermanni* collagen which leads to the carboxylic acid OH stretch becoming the water OH stretch. Moreover, the functional groups of C-Cl and C-Br were also formed via hydrothermal reaction.

**Figure 2:**
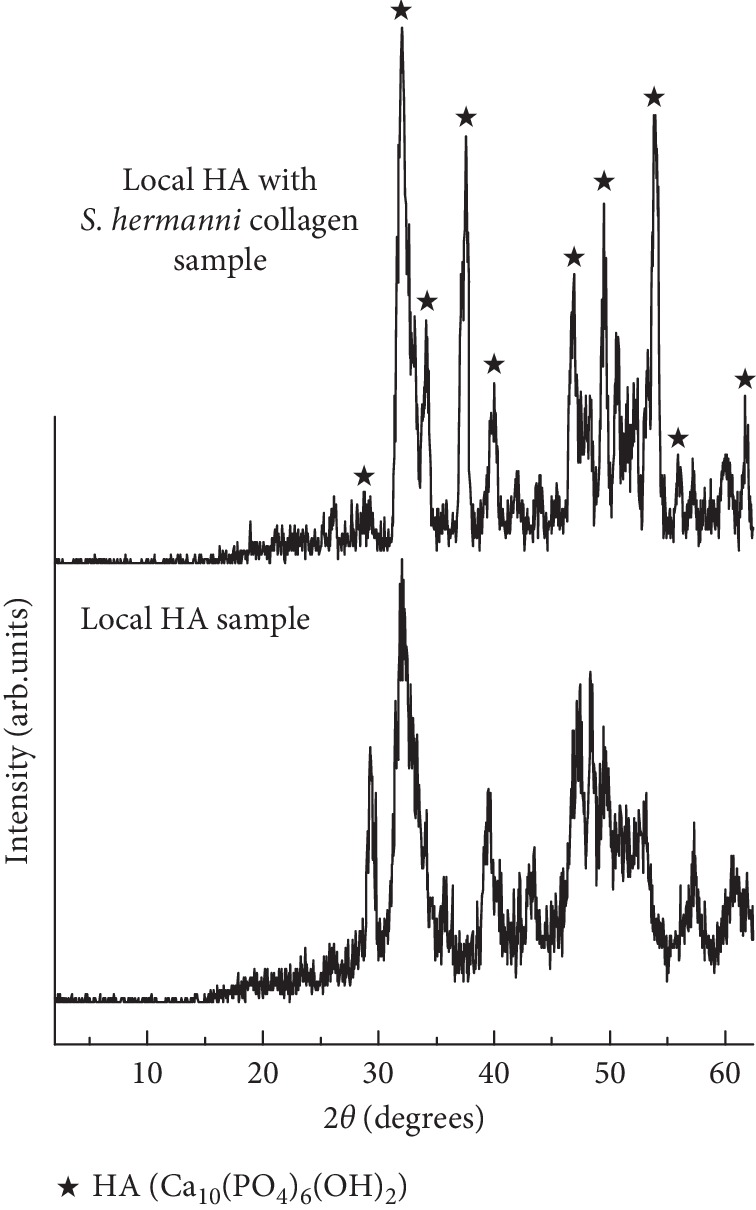
XRD patterns of the local HA with and without *S*. *hermanni* collagen samples.

**Figure 3:**
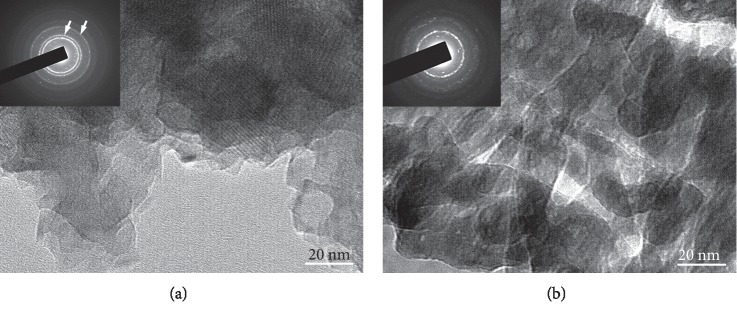
TEM micrographs of (a) the local HA sample and (b) the local HA with the *S*. *hermanni* collagen sample (the ring spots are indicated by white arrows).

Figure 2 displays the XRD patterns taken from the local HA with and without *S*. *hermanni* collagen samples. Obviously, only the diffraction peaks of typical HA (Ca_10_(PO_4_)_6_(OH)_2_) were detected in the local HA sample. After the *S*. *hermanni* collagen addition, similar diffraction results can also be obtained from the local HA with the *S*. *hermanni* collagen sample. Further analyzing by TEM, the ring spots shown by white arrows in the diffraction pattern revealed the existence of the nanocrystalline structure in the local HA sample, as depicted in Figure 3(a). Based on the TEM software analysis, it was verified that the nanocrystalline structure belongs to the HA having a hexagonal close-packed structure. A similar HA with the nanocrystalline structure can also be discovered in the local HA with the *S*. *hermanni* collagen sample (Figure 3(b)). Thus, the addition of *S*. *hermanni* collagen did not influence the microstructure variation of the local HA.

### 3.2. Biocompatibility Evaluation

Table 1 presents the average values of MTT absorbance of the investigated biocomposite samples after 24 h of incubation. In the testing group with doses of 0.015 mg/mL, 0.031 mg/mL, 0.062 mg/mL, and 0.125 mg/mL, the absorbance value against tetrazolium salt is greater than that in the control group. In the testing group with a dose of 0.250 mg/mL, the average absorbance value is slightly higher than that in the control group. It was found that the testing group with a dose of 0.062 mg/mL had the highest absorbance value (i.e., living cells) as compared with all testing groups. However, the testing group with doses of 0.500 mg/mL and 1.000 mg/mL exhibited a relative lower absorbance value than the control group. The statistical analysis results showed that there is a significant difference in the MTT absorbance value between the testing group with doses of 0.015 mg/mL, 0.031 mg/mL, 0.062 mg/mL, 0.125 mg/mL, 0.500 mg/mL, and 1.000 mg/mL and the control group (*p* < 0.05) except the testing group with a dose of 0.250 mg/mL (*p* > 0.05). In Table 2, the results of cell death percentage indicated that the cell death percentage in each testing group is less than 50%. Therefore, the investigated biocomposite material in each dose did not cause cytotoxicity effects on fibroblasts and encouraged the growth of more fibroblasts.

### 3.3. Histological Observation and Analysis

**Figure 4:**
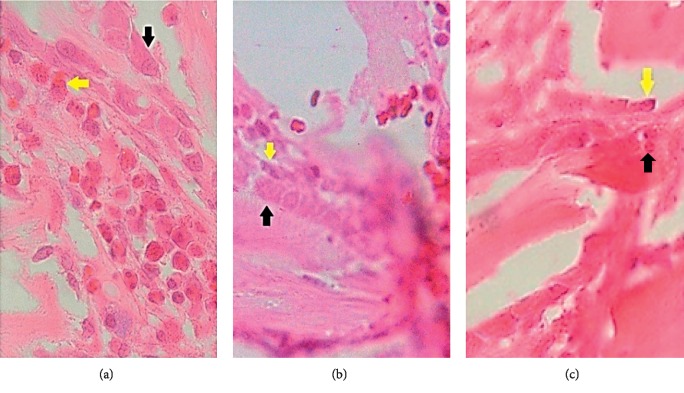
Histological images of the investigated samples after 14 days of healing. (a) Group I: local HA with *S*. *hermanni* collagen. (b) Group II: only *S*. *hermanni* collagen. (c) Group III: only normal collagen (the osteoblasts are indicated by yellow and black arrows).

Table 3 exhibits the average cell numbers of osteoblasts of the investigated samples after different healing periods. It is clearly seen that the investigated biocomposite material sample (group I) showed that osteoblasts started to look visible after day 3. It also happened in group II and group III samples. The number of osteoblasts kept increasing until day 14 and declined on day 28, while the highest number of osteoblasts was observed on day 14. A similar trend can also be found in group II and group III samples. The histological images in Figure 4 reveal the formation of a higher number of osteoblasts (as indicated by yellow and black arrows) in the group I sample as compared with group II and group III samples after 14 days of observation. The analytical results of two-way ANOVA indicated that there is a significant difference between group I, group II, and group III on day 3 (*p* < 0.05), day 7 (*p* < 0.05), day 10 (*p* < 0.05), day 14 (*p* < 0.05), and day 28 (*p* < 0.05). Further analysis via the LSD test showed that there is a significant difference in the osteoblast number in all groups (*p* < 0.05).

## 4. Discussion

In the cytotoxicity evaluation, it was found that the cell death percentage in each testing group is less than 50%. These testing groups did not cause toxicity effects on fibroblasts after 24 h of incubation since the toxicity limit of a substance in the cell culture is 100 *μ*g/mL, which means if a dose of 100 *μ*g/mL did not cause 50% or more cell death, then the substance is considered nontoxic [24]. Accordingly, the fibroblast cells incubated with the highest dose of 1 mg/mL (15.96%) showed no cause of cell death more than 50%. This finding proved that the local HA combined with *S*. *hermanni* collagen possessed good biocompatibility. In addition, the results in Table 3 also found that the number of osteoblasts is highest in the three groups of applications on day 14. The statistical analysis results indicated that there is a significant difference between the investigated samples, healing periods, and interaction (*p* < 0.05). In group I, the number of osteoblasts is higher than that in group II and group III. This characteristic demonstrated that the group of local HA combined with *S*. *hermanni* collagen could increase osteoblast formation. According to previous research by Cotran et al. [25], new bone formation or remodeling occurred in the early phase of osteoid formation through osteoblast activities that synthesized collagen, while the synthesized collagen deposited on an organic mineral phase will cause cell binding, cell proliferation, cell differentiation, and extracellular matrix forming, thus prompting the formation of calcification of new bone tissue [25, 26].

Furthermore, it was found that the success of bone remodeling is based on the increased number and activity of osteoblasts on day 7 and day 10. This is consistent with the statement from the report by Katagiri and Takahashi [27] which stated that cell bonding, cell proliferation, and cell differentiation occur on the first week. A similar result on the increased number of osteoblasts and osteoclasts on day 7 and day 14 can be found in the previous study [28]. The findings also demonstrated that the investigated biocomposite material promotes more osteoblast formation because of high type I collagen fiber composition on *S*. *hermanni* collagen. The *S*. *hermanni* collagen extracts contain higher collagen fibers as compared with other groups [18, 19]. Osteoblast requires amino acid to synthesize collagen for bone basic material. Amino acid is obtained from the biodegradation process of collagen fibers [25]. In the calcification phase, the calcium salt is needed for the calcification material that can be obtained from HA to harden the new bone [29]. However, the gypsum (calcium sulfate) itself played a vital role in the new bone formation via the osteoinduction property in the investigated biocomposite material. Especially for the *α*-calcium sulfate hemihydrate (*α*-CSH (CaSO_4_·0.5H_2_O)), it has been extensively used as a bone graft substitute material because of its high self-setting strength, excellent osteoinduction characteristics, biocompatibility, and angiogenesis properties [30, 31]. A similar fabrication method using a microwave system to generate *α*-CSH proved that the microwave-synthesized *α*-CSH not only induces angiogenesis formation but also facilitates osteogenesis [9]. Based on a new strategic development of the bone graft material, the microwave-synthesized *α*-CSH is a promising biomaterial that can be added in the investigated material as the novel biocomposite bone graft material in the future.

## 5. Conclusion

The local HA with a hexagonal close-packed structure can be synthesized from gypsum powder using hydrothermal reaction, and its microstructure and functional groups did not change by the addition of extracted *S*. *hermanni* collagen. The investigated biocomposite material is nontoxic and biocompatible. The material could promote osteoblast formation and thus enhance the rate of bone healing. Therefore, the local HA combined with extracted *S*. *hermanni* collagen is a promising biomaterial for biomedical applications.

## Figures and Tables

**Table 1:** Average values of MTT absorbance of the investigated biocomposite samples at different concentrations (mg/mL) after 24 h of incubation.

Control	The investigated biocomposite material						
Blank	1.000^*∗*^ (mg/mL)	0.500^*∗*^ (mg/mL)	0.250^*∗*^ (mg/mL)	0.125^*∗*^ (g/mL)	0.062^*∗*^ (mg/mL)	0.031^*∗*^ (mg/mL)	0.015^*∗*^ (mg/mL)
1.538 ± 0.0003	1.264 ± 0.0090	1.348 ± 0.0054	1.539 ± 0.0092	1.594 ± 0.0090	1.688 ± 0.0043	1.607 ± 0.0002	1.586 ± 0.0026

Data are presented as mean ± SD. ^*∗*^*p* < 0.05.

**Table 2:** Cell death percentage of the investigated biocomposite samples at different concentrations (mg/mL) after 24 h of incubation.

Control	The investigated biocomposite material						
Blank	1.000 (mg/mL)	0.500 (mg/mL)	0.250 (mg/mL)	0.125 (mg/mL)	0.062 (mg/mL)	0.031 (mg/mL)	0.015 (mg/mL)
%	15.96	12.43	1.24	−4.31	−6.54	−5.82	−3.14

Data are presented as mean ± SD.

**Table 3:** Average cell numbers of osteoblasts of the investigated samples after different healing periods.

Healing period (day)	3	7	10	14	28
Group I	9,1400^*∗*^ ± 0.8176	12,9810^*∗*^ ± 1.8342	16,3510^*∗*^ ± 1.3756	35,6520^*∗*^ ± 0.7216	28,2450^*∗*^ ± 0.2107
Group II	8,1610^*∗*^ ± 0.9230	11,3220^*∗*^ ± 0.9142	13,8420^*∗*^ ± 0.8588	34,1610^*∗*^ ± 1.2284	24,8410^*∗*^ ± 0.85120
Group III	6,3910^*∗*^ ± 0.3721	10,1620^*∗*^ ± 1.5312	12,4420^*∗*^ ± 0.6717	28,0650^*∗*^ ± 0.61315	21,1410^*∗*^ ± 0.5296

Data are presented as mean ± SD. ^*∗*^*p* < 0.05.

## Data Availability

The data used to support the findings of this study are available from the corresponding author upon request.
